# Organs-on-chip technology: a tool to tackle genetic kidney diseases

**DOI:** 10.1007/s00467-022-05508-2

**Published:** 2022-03-14

**Authors:** Marta G. Valverde, João Faria, Elena Sendino Garví, Manoe J. Janssen, Rosalinde Masereeuw, Silvia M. Mihăilă

**Affiliations:** grid.5477.1 0000000120346234 Div. Pharmacology, Utrecht Institute for Pharmaceutical Sciences, Utrecht University, Utrecht, the Netherlands

**Keywords:** Genetic kidney disorders, Gene editing, In vitro models, Organ-on-chip, Kidney-on-chip

## Abstract

Chronic kidney disease (CKD) is a major healthcare burden that takes a toll on the quality of life of many patients. Emerging evidence indicates that a substantial proportion of these patients carry a genetic defect that contributes to their disease. Any effort to reduce the percentage of patients with a diagnosis of nephropathy heading towards kidney replacement therapies should therefore be encouraged. Besides early genetic screenings and registries, in vitro systems that mimic the complexity and pathophysiological aspects of the disease could advance the screening for targeted and personalized therapies. In this regard, the use of patient-derived cell lines, as well as the generation of disease-specific cell lines via gene editing and stem cell technologies, have significantly improved our understanding of the molecular mechanisms underlying inherited kidney diseases. Furthermore, organs-on-chip technology holds great potential as it can emulate tissue and organ functions that are not found in other, more simple, in vitro models. The personalized nature of the chips, together with physiologically relevant read-outs, provide new opportunities for patient-specific assessment, as well as personalized strategies for treatment. In this review, we summarize the major kidney-on-chip (KOC) configurations and present the most recent studies on the in vitro representation of genetic kidney diseases using KOC-driven strategies.

## Introduction

Genetic kidney diseases are a major cause of chronic kidney disease (CKD) development and account for the majority of pediatric cases and ~ 10% of adult cases in need of kidney replacement therapy [1]. With the latest technological advances, like next generation sequencing and genome-wide association studies, it is expected that even more genetic cases will be identified. This is good news as in a majority of the cases a genetic diagnosis helps patients and doctors to gain insight into disease prognosis, treatment, or transplant decisions [2]. However, in many cases, there is still no therapy available to halt the disease progression and patients rely on kidney replacement therapy for their survival.

The kidney is a complex organ, home to multiple cell types with specific roles and functionalities. Their coordinated activity enables the clearance of metabolic waste products and balancing fluid and blood electrolytes. The nephron, the functional unit of the kidney, is comprised of different structural segments, each with its own assigned function. Every segment is susceptible to genetic mutations, with the majority of the genetic kidney disorders manifesting as glomerulopathies and tubulopathies. These disorders result in an overall malfunction of the kidney with life-threatening consequences. Although next-generation sequencing technologies can promote the clinical understanding of these genetic kidney diseases by providing guidance for molecular diagnostics and personalized treatment, this alone will not suffice. The combination of next-generation sequencing data and appropriate disease modeling will most likely provide a detailed vision of the complex genetic etiology. This was previously shown in a study where researchers found a variant of the *PKHD1* gene, of which mutations cause polycystic kidney disease (PKD), and through in vitro testing of patient-derived cells were able to find ciliary defects in these cells, which were not detected earlier [3].

The development of disease models to understand the mechanisms of human genetic kidney disorders is of great interest. However, meeting the specificities of each disorder when proposing a model is not a trivial task. A plethora of disease models, ranging from zebrafish and *C. elegans* to rodents and in vitro patient-derived cell culturing, have contributed to the identification of novel genetic causes, new therapeutic targets, and to the development of new treatments. For a better overview of these models, we guide the reader to the work of Molinari et al. [4].

Although the ease of manipulation of in vitro cellular models allows the straightforward dissection of disease molecular mechanisms, the complexity of in vivo (animal) models supports the study of multiple cell interactions and tissue homeostasis in a (patho)physiologic context. Nevertheless, animal models do not fully genocopy or phenocopy human disease due to interspecies differences. They are too physiologically complex for a minimal reconstruction approach. The heterogeneity and the low frequency of certain kidney disorders prompt for the quest of more accurate and human patient-specific disease models [5]. With the current developments in (bio)manufacturing and the robust isolation and generation of disease-specific cells and organoids, in vitro disease models based on organs-on-chip (OOC) technology have been pushed to the forefront of scientific discoveries. OOC technology refers to microfluidic cell culture systems with controlled, dynamic conditions that directly replicate the microenvironment of tissues in the human body. OOC-based approaches are beyond traditional, flat, 2D cultures, as they exhibit tissue- and organ- level functions that are not found in other, more simple, in vitro models. Due to their physiologically relevant read-outs, OOC are used for preclinical drug testing and, more recently, as controlled, miniature representation of specific patients and diseases [5, 6]. In this review, we will first consider the major traits of genetic kidney diseases and discuss the cell-based in vitro models for their study. In this context, we will provide an overview of the current advances in the development of OOC-based in vitro models towards studying genetic kidney disease and discuss their potential use as tools to facilitate further insights into disease pathomechanisms and development of new therapeutical interventions.

## Genetic kidney diseases

**Fig. 1:**
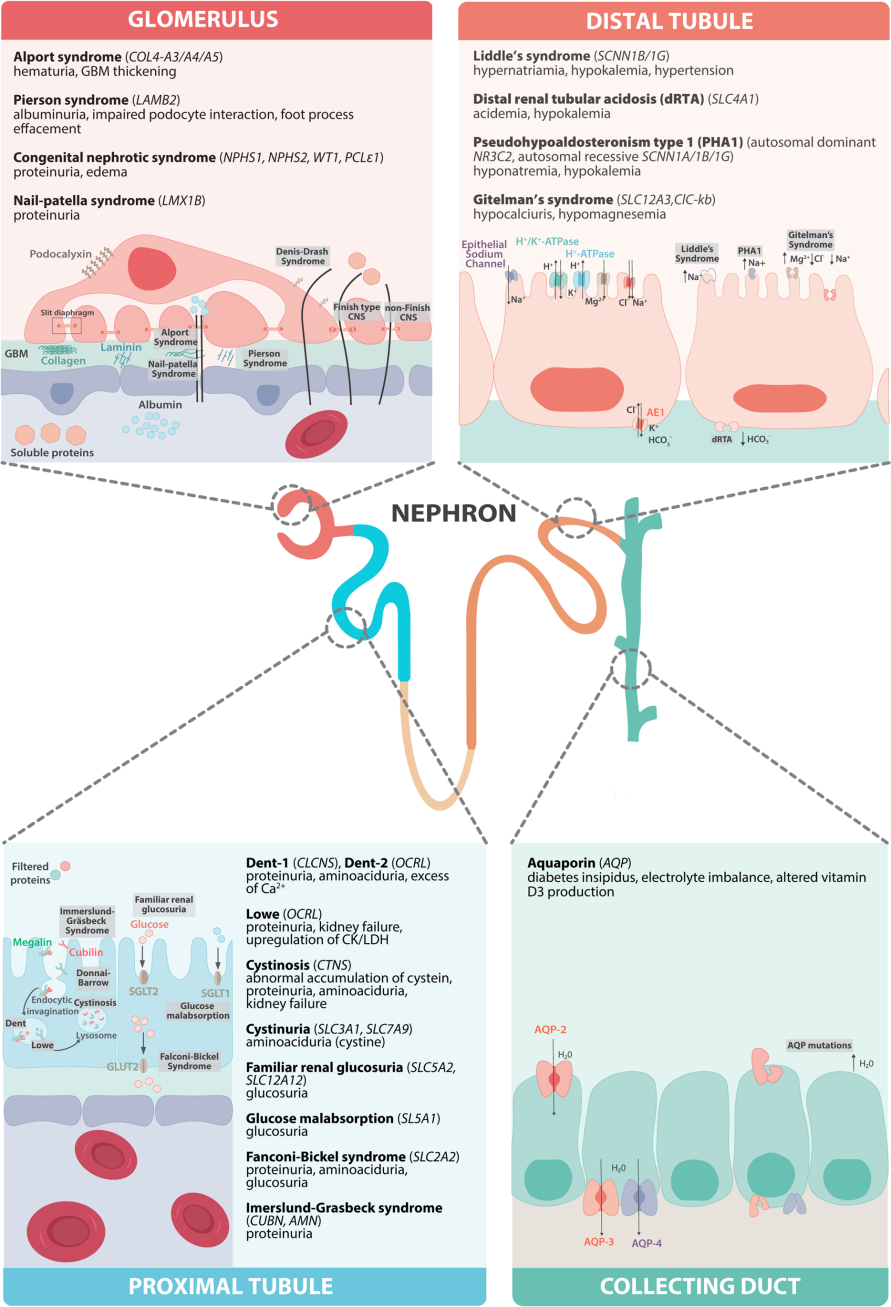
Overview of the different segments of the nephron and their associated kidney genetic diseases. Glomerulus: congenital nephrotic syndrome (CNS) groups the genetic disorders that affect key glomerular filtration barrier (GFB) structural proteins. Slit diaphragms are affected by mutations in the genes *NPHS1* (Finnish type CNS), *NPHS2* (non-Finnish CNS) and *WT1* (Denys-Drash syndrome) [7][7]. Within the GBM, the genetic disorders alter the encoding of collagen IV isoforms (Alport Syndrome, AS) [37], laminin (Pierson syndrome) [38], and *LMX1B* (less known, nail patella syndrome) [35]. Proximal tubule: formed by ciliated epithelial cells that are in close contact with capillaries. On the one hand, most of the disorders affecting the proximal tubule derive from aberrations in genes encoding for transporters of calcium, sodium, and potassium [50]. For instance, mutations in the genes *CLCN5*, *OCRL1*, causing Dent disease 1 and 2, respectively. Additionally, mutations in *OCRL* develop into Lowe syndrome [51]. On the other hand, mutations in genes encoding for key components in metabolic pathways are also associated with proximal tubulopathies. Defects in the *CTNS* gene lead to an abnormal intra-lysosomal accumulation of cystine that causes cystinosis and acidosis, which could eventually lead to Fanconi Syndrome and kidney failure. Distal tubule: the distal tubule epithelial cells express epithelial sodium channel (ENaC) and potassium channels in the apical membrane. Thus, genetic diseases often affect the regulation of Na–K reabsorption [60]. Mutations in the genes *SCNN1B* or *SNCN1G* have been linked to Liddle syndrome, characterized by excessive Na + reabsorption [67]. distal renal tubular acidosis (dRTA) is rooted in mutations in *SLC4A1* [68]. The dominant pseudohypoaldosteronism type 1 (PHA1) is associated with *NR3C3* mutations whilst recessive refers to *SCNN1A*, *1B* and *1G* [69]. Magnesium transporters are also subjected to genetic diseases, such as Bartter syndrome and its variant, Gitelman syndrome. While the former appears in the ascending limb of Henle’s loop, the latter is localized in the distal convoluted tube [70]. Gitelman has been associated with mutations in the *SLC12A3* gene, which codes for the sodium-chloride cotransporter, and with *CLCNKB* gene, coding for renal chloride channel ClC-kb [70]. Collecting duct: the last segment of the nephron is in charge of water and Na + reabsorption. Aquaporin channels (*AQP2*) are also susceptible to mutations leading to dysfunction [72]

A large number of human genetic diseases (> 150) that affect kidney development or kidney tissue maintenance lead to functional and structural defects [1], as observed in most cases of CKD [7]. The range of phenotypes associated with genetic aberrations is remarkably wide. The renal cells that are most involved in the pathogenesis of various inherited kidney diseases include the following: (1) podocytes, the main performers of the renal glomerular filtration barrier, and (2) the (proximal) tubular epithelial cells of the nephron responsible of secretion, reabsorption and ion exchange [8]. A comprehensive overview of genetic kidney aberrations, their molecular and biological implications and associated pathological presentation has been prepared by others and is beyond the scope of the current review [9, 10]. As a summary, Fig. 1 includes the most common diseases and their altered phenotype throughout the segments of the nephron. The increased acceptance of early genetic testing and the establishment of national and international registries, allow for a real-time overview of genetic, medical, and family history. In this direction, the web-based registry, established by European Rare Kidney Disease Reference Network (ERKNet) (https://www.erknet.org), covers patients with rare kidney diseases, provides epidemiological and therapeutic management information, and includes patient cohorts for clinical research [11]. The in-depth documentation of new phenotypes and the isolation of patient-derived cells, made available to the scientific community, would support the development of disease-specific in vitro representations of the disease toward personalized therapeutic discoveries, with the ultimate goal to delay the onset of early-stage CKD [12].

## In vitro cell-based models of genetic kidney diseases

Several disease models (animal and cellular) for inherited kidney diseases have been developed to identify aberrant pathways and novel therapeutic targets, or as drug screening and testing platforms [4]. Deviating from animal models that are unable to fully recapitulate human pathophysiology, human models are becoming highly appealing [13]. In vitro cell cultures have been shown to be efficiently applied to dissect the molecular pathways of genetic diseases, while providing a fast and cheap platform for high-throughput drug screening. Special attention has been paid to identify new cell sources in such a way that patient-derived cells or cells with a predefined mutation can be tested. Hence, not only can we better understand the pathological manifestations of the disease, but also design targeted and personalized therapies towards the phenotype and/or repairing the defective gene [10]. The easy and non-invasive isolation of podocytes and proximal tubule epithelial cells (PTECs) from patient urine samples can generate primary kidney cell cultures that retain the genetic signature of the disease [10, 14]. However, these cells cannot be cultured indefinitely and/or lose their phenotypical signature and spontaneously dedifferentiate, mainly due to the lack of a kidney-like microenvironment. Via immortalization, their phenotype can be conserved. Similarly, cells can be obtained from patient’s kidney biopsies and subsequently immortalized; however, the invasive retrieval procedure and the lack of availability of kidney material make this approach challenging and less used in practice [10, 15]. Still, immortalized, patient-derived cell lines may lose their sensitivity to external stimuli, such as drugs, becoming a *faux* representation of an otherwise dynamic and highly responsive biological system. Moreover, since these cell lines cannot be generated from every single patient, they are solely a representation of one specific patient, and the extrapolation to a population of distinct and unique individuals is limited.

Using gene editing tools, such as clustered regularly interspaced short palindromic repeat (CRISPR/Cas) technology, a candidate mutation can be precisely introduced in healthy cells, allowing a fast and easy generation of mutation-specific diseased cells, paving the way toward precision medicine [16]. An example includes the recent application of CRISPR/Cas9 on a human PTECs line to generate a model for cystinosis that revealed novel insights into the molecular mechanisms of the disease and the potential therapeutic effect of a new combination treatment to alleviate the symptomatology of the disease [17]. Using the same technology, we can target and modify virtually any cell type. By introducing the correct version of the gene into the genome of diseased cells, reversing the disease is in sight. Despite the versatility and the specificity of CRISPR/Cas technology that make it a powerful tool for the generation of disease models in vitro, this gene-editing tool still harbors some limitations. The editing efficiency of the CRISPR/Cas system differs vastly among various cell types, which hampers the generation of disease models due to high optimization costs and time consuming experiments. Additionally, CRISPR/Cas editing presents relatively high off-target effects, which could result in a misleading disease phenotype if not properly evaluated. Lastly, some cells may be sensitive to the DNA damage induced by CRISPR/Cas which can trigger apoptosis, preventing the generation and further study of these disease models [18].

The discovery of human-induced pluripotent stem cells (hiPSCs) has been instrumental for the growth of the in vitro disease modeling field [19–21]. These indefinitely growing cells are suitable alternatives that overcome ethical concerns related to animal studies and reflect the donor’s genetic background. In short, pluripotency is induced in adult somatic cells using four retrovirally transfected transcription factors (the Yamanaka factors) [22]. Then, hiPSCs can be directed towards the desired cell type by a finely tuned combination of growth factors [23]. Using hiPSCs, it is possible to, theoretically, generate all cell types existing in the kidney, which otherwise cannot be obtained by employing classical isolation strategies. When hiPSCs are isolated from a donor that carries a specific gene mutation, this characteristic will be maintained in the differentiated cells. Furthermore, using healthy hiPSCs and employing gene editing tools, such as CRISPR/Cas, insertions and deletions can be performed, which result in ‘diseased’ kidney cells after differentiation. Creating isogenic models also avoids misinterpretation of the results derived from differences in genetic background among donors. Nevertheless, the fact that these cells possess phenotypic heterogeneity, tumorigenicity derived from undifferentiated iPSCs in the cell population, and above all, inefficient recapitulation of late-onset diseases due to the lack of maturity in iPSC-derived cells limits their application in personalised medicine [24]. To date, glomerular and proximal tubule cells have been successfully generated from hiPSCs [25–27].

Kidney-derived cells from hiPSCs and adult stem cells (ASCs) can also be cultured as 3D self-organized multi-cellular structures, known as organoids, which offer a more in vivo-like representation of the cellular heterogeneity resulting in a superior disease model when compared to 2D cell culture. ASCs isolated from kidney tissue and/or urine were used to generate tubuloids, organoids recapitulating the adult kidney tubular epithelium. These tubuloids have then been thought to be included in a biobank in which different patient-derived tubuloids can be used for the study of inherited kidney tubulopathies [28, 29]. Increasing the repertoire of disease-specific organoids will allow us to unravel the role of genotype in the disease process and whether common or divergent disease mechanisms occur in patients with the same diagnosis but different genetic context. The use of high content analysis methodologies, such as RNA sequencing and proteomics, enable the identification of molecular networks that are altered [30, 31]. Nevertheless, organoid maturation is limited, comparable to embryonic kidney during the first trimester, even if maintained for long periods in culture [32]. Thus, their use to model fully developed kidney diseases is limited. Even more, organoid cultures lack physical directional cues that drive the appropriate self-organization for the cells within the organ. To improve the level of maturity of the organoid system, biomechanical stimulation (via flow) and the introduction of vascular networks (via flow or host implantation) have been proposed. These features are crucial for the complete study of phenotype and tissue- and organ- level manifestation of the disease [33]. The implementation of microfabrication and microfluidic devices, such as OOC, enables the introduction of flow in vitro, allowing a better representation of the in vivo situation [34–36].

## Organs-on-chip: a toolbox to model human diseases

Organs-on-chip, also known as microphysiological systems, merge multiple research fields aiming to recapitulate key functional aspects of organs and tissues. The fundamental principle is to culture cells under fluid dynamic conditions rather than in 2D static culture plates or 3D inert matrices, thereby mimicking the biomechanical stimulation and the movement of fluids that occur within the body. The OOC field has experienced significant advances as it develops concurrently with and incorporates methods for growth and maturation of cells, fast and efficient genome editing methods, high-throughput screening, intricate 3D bioprinting, sophisticated sensors, controlled microfluidics, and microfabrication.

There is a wide variety of configurations, usually adapted to a specific application and organ particularities. However, all OOC have five defining characteristics: (1) the architectural arrangement of tissue-replicate, (2) the culture of multiple cell types to capture cellular interactions, (3) the integration of mechanical cues in the form of flow (blood vessels, urine) and stretch (pulsatile blood flow), (4) sensing, and (5) drug stimulation or delivery.

**Fig. 2:**
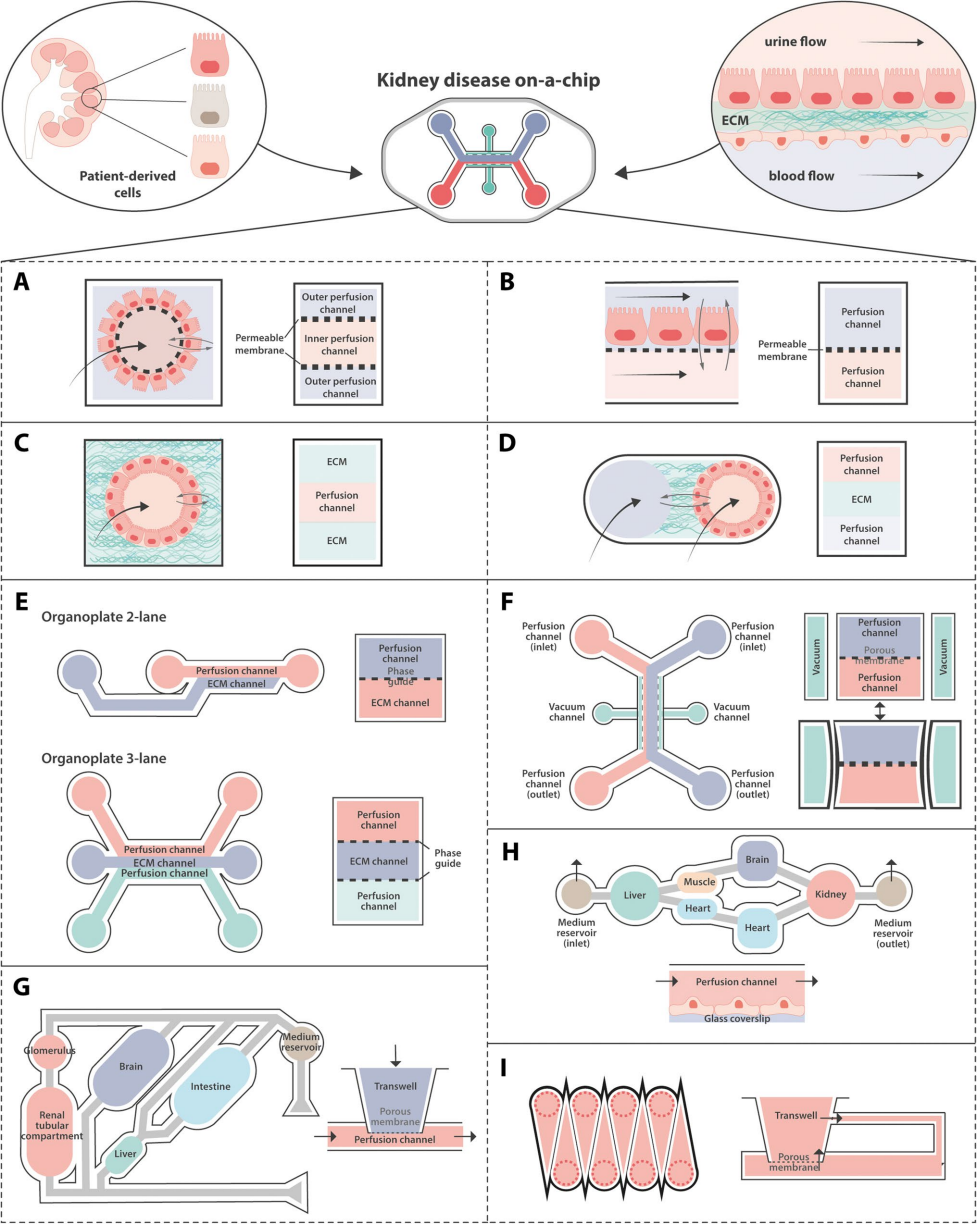
One-size does not fit all. The KOC platform that integrates the mechanical (flow), biochemical (drugs), functional (transport) and microenvironmental (extracellular matrix, ECM) cues to unravel the performance of patient-derived cells. Below, the schematic representation of various OOC devices. The common components of the chips are cells (kidney epithelial in orange and endothelial in purple), microfluidic channels and permeable interfaces (including porous membranes and ECM-like materials (in green)). (**A**) Hollow fiber with an outer monolayer of cells. (**B**) Two channels separated by a porous membrane. Single (**C**) and two (**D**) channels surrounded by ECM-like materials. (**E**–**I**) Schematics of commercially available organs-on-chip applied for kidney-on-chip. (**E**) MIMETAS BV proposes several models, including *Organoplate 2-lane* with two channels and *Organoplate 3-lane 40*, with 3. Instead of a membrane, the adjacent channels are separated by their “PhaseGuide™”, a meniscus forming barrier that allows the direct interaction of the cells with an ECM-filled channel. (**F**) Emulate chips consist of four channels. The two perfusable channels for cell culture are separated by a porous membrane, while the lateral are connected to a vacuum pump that applies stretch forces to the membrane. **G** *Humimic CHIP* by Tissues relies on the attachment of the porous membranes of Transwells to a perfusable channel in order to create a multi-organ-on-a-chip. Similarly, Hesperos (**H**) achieves co-cultures by connecting several wells by means of a continuous perfusion channel. CN Bio Innovations (**I**) uses modified Transwells to culture the cells. MIMETAS BV and CN Bio Innovations use rocking systems to create a bidirectional flow, while Emulate, Tissuse and Hesperos reproduce a uni-directional flow by circulating media from inlet to outlet

The building blocks to develop the most trivial OOC configuration refer to the cells that will populate the chip, the compartmentalization (apical and basal) of the device and the fluid dynamics through the compartments (Fig. 2). With the advance of biofabrication and manufacturing techniques, more sophisticated configurations have been developed; however, in essence, all of them can be reduced to the assembly of the building blocks. Some of the most common OOC configurations are depicted in Fig. 2A–D. In more detail, an OOC consists of a chamber with one or two micrometric channels enclosed by a permeable interface. The inlets/outlets are connected to a perfusion system that allows recirculation of the medium for its posterior analysis. The dynamic microenvironment created in the chip imitates native tissue interfaces and mechanical stimulation by providing shear stress and pulsatile flows in (co-)culture systems. Regarding the permeable dividing interface, hollow fibers, flexible porous flat membranes, and hydrogels are being used. These are specifically designed and customized to allow crosstalk between cells and substance exchange within channels [37]. Fibers and membranes are stiffer, but more stable over time, whereas gels are not as durable. But gels can better mimic the native microenvironment, for instance the tubular interstitium. Additionally, sensors can be incorporated in the system, providing real-time readouts for the control of the cell culture (pH, oxygen levels, temperature [38], monolayer integrity [39–41], molecule absorption [41], cell orientation [42], and shear stress [41]).

Owing to the increased demand for these advanced models, standardized, user-friendly, and ready-to-use alternatives are now available, proving that upscaling and high(er)-throughput are achievable. Looking through the lens of specific context of use, for instance, the study of genetic kidney dysfunctionality related to a particular nephron segment, researchers can opt for customizable platforms. For both commercial (Fig. 2E–I) and custom-made alternatives, the preferred materials for manufacturing the chambers are resins and synthetic polymers, including silicones and thermoplastics. On top of being biocompatible and multipurpose, these materials have relatively low-cost, long shelf-life and can be easily combined with other polymers and hydrogels to increase their cell affinity. These materials also provide a better control of the compounds to be studied as polydimethylsiloxane, the most common material used for the biofabrication of OOCs, absorbs hydrophobic compounds, making it a less suitable material for drug testing [43].

The micrometrical characteristics of OOC devices involve practical difficulties. For tailored devices, the design, manufacturing, and assembly are highly technical and laborious efforts that require facilities and techniques that may not be available for all research groups. Outsourcing the fabrication solves the matter, but increases the costs of the devices. The commercially available alternatives are ready to use. Moreover, training is often provided by the suppliers, easing their acceptance in the field. However, the commercial systems are not customizable and parameters, such as flow directionalities and rates, are fixed and inherent to the chip. Lastly, it is noteworthy that the readouts of OOC are restricted by the reduced number of cells and the limited access to the channels. The majority of the results are limited to imaging techniques, such as immunohistological stainings and permeability of fluorescently labeled molecules, omitting futher genetic and/or molecular cellular analyses. Increased surface areas within the chip would allow further cellular characterization, but forfeit the *micro* features [44–46].

## Kidney genetic diseases-on-chip

The goal of kidney-on-chip (KOC) studies is to provide a platform for a faithful replication of the key kidney functions. The integration of specific mechanical (flow) and microenvironmental (extracellular matrix) cues should aid in the preservation of the geno- and phenotype of the cells and the associated functional performance, thus recreating the pathophysiology of the disease. A “one size fits all” perspective on KOC would limit the applicability of the system and may not represent the disease, or the individual patient, well enough. Nevertheless, not all studies incorporate the latter, mainly due to patient variability and difficulties in securing enough material to establish the models. In part, this can be attributed to the limited and fragmented interactions between KOC researchers and clinicians, or a lack of understanding of the disease to be studied in the KOC, and its potential clinical implications. These disconnections delay the development of robust, validated, and standardized models that can be applied across laboratories, research institutes, hospitals, and (pharmaceutical) companies. The assembly of collaborative and multidisciplinary teams would facilitate the establishment of such models. Recently, the newly established European Organ-on-Chip Society released a roadmap of OOC development that emphasizes the context needs, initiatives, and specific actions to promote OOC adoption across disciplines. Additionally, the harmonization and interconnection of local databases would generate a worldwide library of models, following the example of ERKNet [47].

This review highlights the first studies that have incorporated genetically compromised kidney cells in a KOC system in an attempt to establish a disease model and evaluate drug efficacy. Interestingly, the reviewed articles focus on a specific function of the kidney, and, thus, attempt to replicate a specific region of the nephron, rather than the whole unit. Several in vitro cell models that depict different genetic kidney diseases are available and have been extensively reviewed by Bondue et al*.* [10], however not all have been translated to a KOC platform. Indeed, the in vitro modeling of genetic kidney diseases using KOC is in its infancy, and we believe that the current attempts advocate for an adoption of this approach by the research community at large.

### Glomerulus-on-chip

The first function of the nephron is blood filtration in the renal corpuscle and occurs when solute-laden blood passes across the glomerular filtration barrier (GFB) composed of podocytes, the glomerular basement membrane (GBM) and endothelial cells. On-chip replicas of the GFB have been proposed based on the use of podocytes and endothelial glomerular cells, and their innate ability to secrete the GBM components [48]. Petrosyan et al, used a microfluidic device designed to replicate GFB using different types of podocytes and endothelial cells [49]. Podocytes derived from amniotic fluid of Alport syndrome (AS) patients were also incorporated in the model. Similar to the in vivo situation, the defective GBM produced by the diseased podocytes led to impaired permselectivity for albumin. Exposure to high levels of glucose and to the aminonucleoside puromycin were also assessed, proving the suitability of this model to mimic conditions of diabetic nephropathy and drug-induced nephrotoxicity [49]. Petrosyan’s model was based on the use of a commercially available chip, represented in Fig. 2E. Although the *OrganoPlates®* enable high-throughput studies, the system is limited by the use of collagen I in the intermediate channel (not a native component of the healthy GBM) and bidirectional flows achieved by a 2D rocker. In a similar approach, Iampietro et al. used a microfluidic device to study urine-derived AS podocytes. It was proven that the permeability of diseased podocyte–endothelial cell co-cultures was higher than their healthy counterparts [50]. The same team has recently included in the device podocytes derived from patients carrying apolipoprotein L1 (*APOL1*) high-risk genotypes (HRGs) G1 and G2 [14]. *APOL1* HGR mutations are known for increasing the risk of kidney-related diseases affecting podocyte activity. Under perfusion in the chip, the HRG podocytes exhibited pathological phenotypes, such as altered cytoskeleton and increased permeability [14]. The system used by Levtchenko’s team presents some key advantages since it allows collagen IV coating and unidirectional tunable flow. Moreover, the cells and filtrate of the upper and lower compartments can be recovered, allowing their direct analysis. Diabetic nephropathy [51] and glomerular hypertension [52] models have also been proposed.

Flow shear stress (FSS) has been used to maintain podocyte maturation. Yang et al. proved that the combination of FSS and retinoic acid relented podocyte dedifferentiation, by characterizing the expression of maturation markers (*NPSH1*, *NPSH2* and *WT1*) and cell morphology [53]. KOC has also been used to enhance the differentiation of hiPSC-derived podocytes [26, 27], and to improve the maturation and vascularization of glomerular organoids [54]. In both cases, the glomerular cells exhibited a more mature phenotype, evaluated by the increased expression of signature biomarkers when compared to static conditions, and the formation of a robust barrier.

These advances advocate for the inclusion of patient-derived cells into the KOC models, bringing them a step closer to personalized medicine. In parallel, consistent advances have been achieved by improving the cellular microenvironment of the KOC device to provide relevant architectures for the cell culture [55–58]. Besides the GFB forming cells, supporting cell types are also present in the glomerulus, including mesangial, granular, and macula densa. Although their contribution is key for filtration rate regulation, endocrinal communication, and structural support, they have often been overlooked and only a few models have included mesangial cells [59, 60].

### Tubulopathies-on-chip

Inherited tubulopathies are characterized by impaired function of one or more specific transport molecules. The clinical presentation can range from alterations in the concentration of specific solutes in blood or urine to serious and life-threatening disorders of homeostasis.

#### Proximal tubule

After the initial filtrate is formed, secretion and reabsorption of solutes occurs in the proximal tubule (PT) [61]. PTECs from variable sources have been seeded on chips, proving that the presence of FSS enhanced cell alignment, tight junction formation, and transporter expression [62–65]. Because the PT and vasculature are in close contact, both anatomically and in terms of cell interaction, a two-channel chip is the evident candidate to mimic this interaction. Vedula et al., presented a PT-on-chip comprised of PTECs and microvascular endothelial cells [66]. Positive proliferation feedback loops were established upon co-culturing when compared to their previous works which included only PTECs [65], and glucose reabsorption from tubular lumen to the endothelial site was shown. Vriend et al. applied FSS to explore its effect on cell morphology and transporter activity in the PT [67]. FSS increased the uptake of albumin-FITC, increased transporter activity, improved cell morphology and prompted elongation along the flow path. This was also true for the model that used cilia knockout cells (ciPTEC-KIF3alpha^–/–^), suggesting that apical microvilli are the main mechanotransducers of these cells. Naik et al. have recently developed a 3D microfluidic model in which PT cells were genetically engineered to display the phenotype of Lowe syndrome/Dent disease. In this study, PT cells (HK-2 OCR^–/–^) cultured in the KOC showed shorter cilia when compared to healthy PT cells, which was solved when treated with siRNA. Similarly, the diseased cells showed increased collagen secretion, as seen in Lowe syndrome patients [68].

Another important function of the PT is the activation of vitamin D, important for the reabsorption of calcium [69]. This PT feature has been replicated in a KOC model that showed levels of vitamin D metabolism similar to in vivo, suggesting that these models can improve the maturity and function of PT cells when compared to 2D monolayer cultures [70].

A different approach for perfusable channels was pursued by the Lewis group [71]. By means of bioprinting, two adjacent channels were built in ECM-like substrates. PTECs and glomerular endothelial cells were then seeded in each channel. The same group later demonstrated active reabsorption of solutes, namely albumin and glucose, showing the versatility of this model to mimic both healthy and disease conditions (hyperglycemia) [71]. In this way, this KOC model also incorporated the cells’ interactions with the microenvironment.

#### Distal tubule

Distal tubule (DT) possesses unique electrolyte transporters and, unlike the PT, the microvilli are smaller and less dense resulting in a wider lumen [72–74]. To our knowledge, only Wang et al.particularly focused on developing a DT-on-chip [75]. The team studied the infection with pseudorabies virus on electrolyte transport, particularly the Na^+^–Cl^–^ cotransporter and Na^+^K^+^–ATPase. A successful analysis of the Na^+^ reabsorption on the chip supported the evidence that the beforementioned genetic alterations could be investigated further in this setup. The results proved that cells exposed to FSS showed better Na^+^ reabsorption, tighter cell junctions, more acetylated tubulin expression, and larger cell heights with more microvilli on the apical side, when compared to the static controls [75]. Moreover, this report is the first one of its kind that included viral infections. This proof of concept opens the door to viral transductions on-chip, a field which remained unexplored and could potentially lead to the establishment of more precise in vitro models.

#### Collecting duct

The collecting duct (CD) is the final segment of the nephron and allows urine to be excreted as waste. The main process in this segment is the reabsorption of water and Na^+^ [76]. To date, the only available OOC-based model was proposed by Jang et al. by culturing rat inter-medullary CD cells on a single-channel chip [77]. They established that FSS, the addition of arginine-vasopressin and osmolar differences between channels regulate cytoskeletal organization and aquaporin migration towards the apical domain. Moreover, the migration process was reversed after terminating fluidic stimulation [77].

## The step forward: a combined KOC model

In the last few years, a substantial number of attempts to develop KOC have been proposed, including conceptual approaches based on computational models [78]. Nevertheless, the tendency is to move towards a system of chips in which each segment can be separately replicated and then connected. The combination of glomerular filtration and tubular reabsorption processes was proposed by Sakolish and Mahler who combined a biotic filter, mimicking glomerular filtration, with a PT chip [79]. Even though the glomerular segment lacked podocytes, their data supports that the combination of segments is not only possible, but also suitable to sustain enhanced junctions, baso-apical polarization, cytoskeletal reorganization and up-regulation of transport proteins. KOC has been combined with remote organs, including the liver, to establish systemic models [80]. A “body-on-a-chip” including kidney, intestine, liver, heart, lung, skin, blood–brain barrier, and brain has been recently proposed [81]. The addition of a diseased KOC in an otherwise healthy line-up of organs would provide further insight into how those genetic diseases that are largely limited to the kidney affect the activity of remote organs. Many kidney diseases manifest in other organs as well and, thus, in the “patient-on-a-chip” approach in which cells carrying the specific mutation, but representing different organs, are loaded on tissue-specific chips, the systemic implications of the disease could be recapitulated and therapeutically targeted [54, 79, 82, 83].

## Conclusion

Kidney-on-chip emerges as a powerful tool for the in vitro representation of kidney function as it provides a much-needed dynamic platform for disease modeling and drug testing. From glomerulus to distal tubule, researchers have successfully proposed models for the nephron’s segments focusing on the replication of exchange barriers. Despite promising results and a technology readily and commercially available, the current approaches fall short when mimicking genetic diseases. Novel protocols for harvesting primary cells and differentiation of hiPSCs into individual kidney cell types, combined with gene editing, would shape our understanding of the kidney (patho-)physiology and aid in the development of novel therapies. A step towards the personalized approach has been taken. Accelerating the process is a matter of unifying efforts and strengthening the dialogue between biologists, engineers, geneticists, clinicians, and patients, early in the development of the models. Taken together, the unique capabilities of OOC technology can be used to develop new in vitro models of human genetic disease models.

## References

[1] Devuyst O, Knoers NV, Remuzzi G, Schaefer F (2014). Rare inherited kidney diseases: challenges, opportunities, and perspectives. Lancet.

[2] Besse W (2020) Genetic analysis in kidney disease: advancing clinical diagnosis and research discovery. Kidney360 1:720–723. 10.34067/kid.0003632020 10.34067/KID.0003632020 PMC8317592 34327334

[3] Molinari E, Srivastava S, Dewhurst RM, Sayer JA (2020). Use of patient derived urine renal epithelial cells to confirm pathogenicity of PKHD1 alleles. BMC Nephrol.

[4] Molinari E, Sayer JA (2020). Disease modeling to understand the pathomechanisms of human genetic kidney disorders. Clin J Am Soc Nephrol.

[5] van den Berg A, Mummery CL, Passier R, van der Meer AD (2019). Personalised organs-on-chips: functional testing for precision medicine. Lab Chip.

[6] Ashammakhi N, Wesseling-Perry K, Hasan A, Elkhammas E (2018). Kidney-on-a-chip: untapped opportunities. Kidney Int.

[7] Leeuwis JW, Nguyen TQ, Dendooven A, Kok RJ (2010). Targeting podocyte-associated diseases. Adv Drug Deliv Rev.

[8] Downie ML, Lopez Garcia SC, Kleta R, Bockenhauer D (2021). Inherited tubulopathies of the kidney: insights from genetics. Clin J Am Soc Nephrol.

[9] Hildebrandt F (2010). Genetic kidney diseases. Lancet.

[10] Bondue T, Arcolino FO, Veys KRP, Adebayo OC (2021). Urine-derived epithelial cells as models for genetic kidney diseases. Cells.

[11] Bassanese G, Wlodkowski T, Servais A, Heidet L (2021). The European Rare Kidney Disease Registry (ERKReg): objectives, design and initial results. Orphanet J Rare Dis.

[12] Stokman MF, Renkema KY, Giles RH, Schaefer F (2016). The expanding phenotypic spectra of kidney diseases: insights from genetic studies. Nat Rev Nephrol.

[13] Faria J, Ahmed S, Gerritsen KGF, Mihaila SM (2019). Kidney-based in vitro models for drug-induced toxicity testing. Arch Toxicol.

[14] Ekulu PM, Adebayo OC, Decuypere JP, Bellucci L (2021). Novel human podocyte cell model carrying G2/G2 APOL1 high-risk genotype. Cells.

[15] Little MH, Kumar SV, Forbes T (2019). Recapitulating kidney development: Progress and challenges. Semin Cell Dev Biol.

[16] WareJoncas Z, Campbell JM, Martinez-Galvez G, Gendron WAC (2018). Precision gene editing technology and applications in nephrology. Nat Rev Nephrol.

[17] Jamalpoor A, van Gelder CA, Yousef Yengej FA, Zaal EA et al (2021) Cysteamine-bicalutamide combination therapy corrects proximal tubule phenotype in cystinosis. EMBO Mol Med 13:e13067. 10.15252/emmm.202013067 10.15252/emmm.202013067 PMC8261496 34165243

[18] Yang Y, Xu J, Ge S, Lai L (2021). CRISPR/Cas: advances, limitations, and applications for precision cancer research. Front Med (Lausanne).

[19] Zhou T, Benda C, Duzinger S, Huang Y (2011). Generation of induced pluripotent stem cells from urine. J Am Soc Nephrol.

[20] Zhou T, Benda C, Dunzinger S, Huang Y (2012). Generation of human induced pluripotent stem cells from urine samples. Nat Protoc.

[21] Mulder J, Sharmin S, Chow T, Rodrigues DC (2020). Generation of infant- and pediatric-derived urinary induced pluripotent stem cells competent to form kidney organoids. Pediatr Res.

[22] Takahashi K, Tanabe K, Ohnuki M, Narita M (2007). Induction of pluripotent stem cells from adult human fibroblasts by defined factors. Cell.

[23] Low JH, Li P, Chew EGY, Zhou B (2019). Generation of human PSC-derived kidney organoids with patterned nephron segments and a de novo vascular network. Cell Stem Cell.

[24] Doss MX, Sachinidis A (2019). Current challenges of iPSC-based disease modeling and therapeutic implications. Cells.

[25] Roye Y, Bhattacharya R, Mou X, Zhou Y (2021). A personalized glomerulus chip engineered from stem cell-derived epithelium and vascular endothelium. Micromachines (Basel).

[26] Musah S, Mammoto A, Ferrante TC, Jeanty SSF (2017). Mature induced-pluripotent-stem-cell-derived human podocytes reconstitute kidney glomerular-capillary-wall function on a chip. Nat Biomed Eng.

[27] Musah S, Dimitrakakis N, Camacho DM, Church GM (2018). Directed differentiation of human induced pluripotent stem cells into mature kidney podocytes and establishment of a Glomerulus Chip. Nat Protoc.

[28] Schutgens F, Rookmaaker M, Verhaar M (2021). A perspective on a urine-derived kidney tubuloid biobank from patients with hereditary tubulopathies. Tissue Eng Part C Methods.

[29] Schutgens F, Rookmaaker MB, Margaritis T, Rios A (2019). Tubuloids derived from human adult kidney and urine for personalized disease modeling. Nat Biotechnol.

[30] Taguchi A, Nishinakamura R (2017). Higher-order kidney organogenesis from pluripotent stem cells. Cell Stem Cell.

[31] Little MH, Howden SE, Lawlor KT, Vanslambrouck JM (2022). Determining lineage relationships in kidney development and disease. Nat Rev Nephrol.

[32] Wu H, Uchimura K, Donnelly EL, Kirita Y (2018). Comparative analysis and refinement of human PSC-derived kidney organoid differentiation with single-cell transcriptomics. Cell Stem Cell.

[33] Nishinakamura R (2019). Human kidney organoids: progress and remaining challenges. Nat Rev Nephrol.

[34] Romero-Guevara R, Ioannides A, Xinaris C (2020). Kidney organoids as disease models: strengths, weaknesses and perspectives. Front Physiol.

[35] Gijzen L, Yousef Yengej FA, Schutgens F, Vormann MK (2021). Culture and analysis of kidney tubuloids and perfused tubuloid cells-on-a-chip. Nat Protoc.

[36] Wiraja C, Mori Y, Ichimura T, Hwang J (2021). Nephrotoxicity assessment with human kidney tubuloids using spherical nucleic acid-based mRNA nanoflares. Nano Lett.

[37] Quiros-Solano WF, Gaio N, Stassen O, Arik YB (2018). Microfabricated tuneable and transferable porous PDMS membranes for Organs-on-Chips. Sci Rep.

[38] Clarke GA, Hartse BX, Niaraki Asli AE, Taghavimehr M (2021). Advancement of sensor integrated organ-on-chip devices. Sensors (Basel).

[39] van der Helm MW, Odijk M, Frimat JP, van der Meer AD (2016). Direct quantification of transendothelial electrical resistance in organs-on-chips. Biosens Bioelectron.

[40] Henry OYF, Villenave R, Cronce MJ, Leineweber WD (2017). Organs-on-chips with integrated electrodes for trans-epithelial electrical resistance (TEER) measurements of human epithelial barrier function. Lab Chip.

[41] Booth R, Noh S, Kim H (2014). A multiple-channel, multiple-assay platform for characterization of full-range shear stress effects on vascular endothelial cells. Lab Chip.

[42] Rothbauer M, Ertl P (2020). Emerging biosensor trends in organ-on-a-chip. Adv Biochem Eng Biotechnol.

[43] Toepke MW, Beebe DJ (2006). PDMS absorption of small molecules and consequences in microfluidic applications. Lab Chip.

[44] Probst C, Schneider S, Loskill P (2018). High-throughput organ-on-a-chip systems: Current status and remaining challenges. Current Opinion in Biomedical Engineering.

[45] Low LA, Mummery C, Berridge BR, Austin CP (2021). Organs-on-chips: into the next decade. Nat Rev Drug Discov.

[46] Tajeddin A, Mustafaoglu N (2021). Design and fabrication of organ-on-chips: promises and challenges. Micromachines (Basel).

[47] Mastrangeli M, Millet S, Mummery C, Loskill P et al (2019) Building blocks for a European Organ-on-Chip roadmap. ALTEX 36:481–492. 10.14573/altex.1905221 10.14573/altex.1905221 31329263

[48] Valverde MG, Mille LS, Figler KP, Cervantes E (2022). Biomimetic models of the glomerulus. Nat Rev Nephrol.

[49] Petrosyan A, Cravedi P, Villani V, Angeletti A (2019). A glomerulus-on-a-chip to recapitulate the human glomerular filtration barrier. Nat Commun.

[50] Iampietro C, Bellucci L, Arcolino FO, Arigoni M (2020). Molecular and functional characterization of urine-derived podocytes from patients with Alport syndrome. J Pathol.

[51] Wang L, Tao T, Su W, Yu H (2017). A disease model of diabetic nephropathy in a glomerulus-on-a-chip microdevice. Lab Chip.

[52] Zhou M, Zhang X, Wen X, Wu T (2016). Development of a functional glomerulus at the organ level on a chip to mimic hypertensive nephropathy. Sci Rep.

[53] Yang SH, Choi JW, Huh D, Jo HA (2017). Roles of fluid shear stress and retinoic acid in the differentiation of primary cultured human podocytes. Exp Cell Res.

[54] Homan KA, Gupta N, Kroll KT, Kolesky DB (2019). Flow-enhanced vascularization and maturation of kidney organoids in vitro. Nat Methods.

[55] Xie R, Korolj A, Liu C, Song X (2020). h-FIBER: microfluidic topographical hollow fiber for studies of glomerular filtration barrier. ACS Cent Sci.

[56] Tuffin J, Burke M, Richardson T, Johnson T (2019). A composite hydrogel scaffold permits self-organization and matrix deposition by cocultured human glomerular cells. Adv Healthc Mater.

[57] Slater SC, Beachley V, Hayes T, Zhang D (2011). An in vitro model of the glomerular capillary wall using electrospun collagen nanofibres in a bioartificial composite basement membrane. PLoS ONE.

[58] Li Z, Tuffin J, Lei IM, Ruggeri FS (2018). Solution fibre spinning technique for the fabrication of tuneable decellularised matrix-laden fibres and fibrous micromembranes. Acta Biomater.

[59] Waters JP, Richards YC, Skepper JN, Southwood M (2017). A 3D tri-culture system reveals that activin receptor-like kinase 5 and connective tissue growth factor drive human glomerulosclerosis. J Pathol.

[60] Wang PC, Takezawa T (2005). Reconstruction of renal glomerular tissue using collagen vitrigel scaffold. J Biosci Bioeng.

[61] Soo JY, Jansen J, Masereeuw R, Little MH (2018). Advances in predictive in vitro models of drug-induced nephrotoxicity. Nat Rev Nephrol.

[62] Vormann MK, Gijzen L, Hutter S, Boot L (2018). Nephrotoxicity and kidney transport assessment on 3D perfused proximal tubules. AAPS J.

[63] Maass C, Sorensen NB, Himmelfarb J, Kelly EJ (2019). Translational assessment of drug-induced proximal tubule injury using a kidney microphysiological system. CPT Pharmacometrics Syst Pharmacol.

[64] Jang KJ, Mehr AP, Hamilton GA, McPartlin LA (2013). Human kidney proximal tubule-on-a-chip for drug transport and nephrotoxicity assessment. Integr Biol (Camb).

[65] Frohlich EM, Zhang X, Charest JL (2012). The use of controlled surface topography and flow-induced shear stress to influence renal epithelial cell function. Integr Biol (Camb).

[66] Vedula EM, Alonso JL, Arnaout MA, Charest JL (2017). A microfluidic renal proximal tubule with active reabsorptive function. PLoS ONE.

[67] Vriend J, Peters JGP, Nieskens TTG, Skovronova R (2020). Flow stimulates drug transport in a human kidney proximal tubule-on-a-chip independent of primary cilia. Biochim Biophys Acta Gen Subj.

[68] Naik S, Wood AR, Ongenaert M, Saidiyan P (2021). A 3D renal proximal tubule on chip model phenocopies Lowe syndrome and Dent II disease tubulopathy. Int J Mol Sci.

[69] Oliveira B, Unwin R, Walsh SB (2019). Inherited proximal tubular disorders and nephrolithiasis. Urolithiasis.

[70] Weber EJ, Chapron A, Chapron BD, Voellinger JL (2016). Development of a microphysiological model of human kidney proximal tubule function. Kidney Int.

[71] Lin NYC, Homan KA, Robinson SS, Kolesky DB (2019). Renal reabsorption in 3D vascularized proximal tubule models. Proc Natl Acad Sci U S A.

[72] Trepiccione F, Prosperi F, de la Motte LR, Hubner CA (2017). New findings on the pathogenesis of distal renal tubular acidosis. Kidney Dis (Basel).

[73] Furgeson SB, Linas S (2010). Mechanisms of type I and type II pseudohypoaldosteronism. J Am Soc Nephrol.

[74] Knoers NV (2006). Gitelman syndrome. Adv Chronic Kidney Dis.

[75] Wang J, Wang C, Xu N, Liu ZF (2019). A virus-induced kidney disease model based on organ-on-a-chip: pathogenesis exploration of virus-related renal dysfunctions. Biomaterials.

[76] Jung HJ, Kwon TH (2016). Molecular mechanisms regulating aquaporin-2 in kidney collecting duct. Am J Physiol Renal Physiol.

[77] Jang KJ, Cho HS, Kang DH, Bae WG (2011). Fluid-shear-stress-induced translocation of aquaporin-2 and reorganization of actin cytoskeleton in renal tubular epithelial cells. Integr Biol (Camb).

[78] Weinberg E, Kaazempur-Mofrad M, Borenstein J (2008). Concept and computational design for a bioartificial nephron-on-a-chip. Int J Artif Organs.

[79] Sakolish Courtney M, Mahler GJ (2017). A novel microfluidic device to model the human proximal tubule and glomerulus. RSC Adv.

[80] Theobald J, Abu El Maaty MA, Kusterer N, Wetterauer B (2019). In vitro metabolic activation of vitamin D3 by using a multi-compartment microfluidic liver-kidney organ on chip platform. Sci Rep.

[81] Novak R, Ingram M, Marquez S, Das D (2020). Robotic fluidic coupling and interrogation of multiple vascularized organ chips. Nat Biomed Eng.

[82] Borestrom C, Jonebring A, Guo J, Palmgren H (2018). A CRISP(e)R view on kidney organoids allows generation of an induced pluripotent stem cell-derived kidney model for drug discovery. Kidney Int.

[83] Sakolish CM, Philip B, Mahler GJ (2019). A human proximal tubule-on-a-chip to study renal disease and toxicity. Biomicrofluidics.

